# Hedgehog signaling is required at multiple stages of zebrafish tooth development

**DOI:** 10.1186/1471-213X-10-119

**Published:** 2010-11-30

**Authors:** William R Jackman, James J Yoo, David W Stock

**Affiliations:** 1 Department of Biology, Bowdoin College, Brunswick, ME, 04011, USA; 2 Department of Ecology and Evolutionary Biology, University of Colorado, Boulder, CO, 80309, USA

## Abstract

**Background:**

The accessibility of the developing zebrafish pharyngeal dentition makes it an advantageous system in which to study many aspects of tooth development from early initiation to late morphogenesis. In mammals, hedgehog signaling is known to be essential for multiple stages of odontogenesis; however, potential roles for the pathway during initiation of tooth development or in later morphogenesis are incompletely understood.

**Results:**

We have identified mRNA expression of the hedgehog ligands *shha *and the receptors *ptc1 *and *ptc2 *during zebrafish pharyngeal tooth development. We looked for, but did not detect, tooth germ expression of the other known zebrafish hedgehog ligands *shhb, dhh*, *ihha*, or *ihhb*, suggesting that as in mammals, only Shh participates in zebrafish tooth development. Supporting this idea, we found that morphological and gene expression evidence of tooth initiation is eliminated in *shha *mutant embryos, and that morpholino antisense oligonucleotide knockdown of *shha*, but not *shhb*, function prevents mature tooth formation. Hedgehog pathway inhibition with the antagonist compound cyclopamine affected tooth formation at each stage in which we applied it: arresting development at early stages and disrupting mature tooth morphology when applied later. These results suggest that hedgehog signaling is required continuously during odontogenesis. In contrast, over-expression of *shha *had no effect on the developing dentition, possibly because *shha *is normally extensively expressed in the zebrafish pharyngeal region.

**Conclusion:**

We have identified previously unknown requirements for hedgehog signaling for early tooth initiation and later morphogenesis. The similarity of our results with data from mouse and other vertebrates suggests that despite gene duplication and changes in the location of where teeth form, the roles of hedgehog signaling in tooth development have been largely conserved during evolution.

## Background

The hedgehog pathway is an evolutionarily ancient cell signaling system shared among all metazoans [1,2]. While extensively studied [3], recent work continues to highlight the essential role of this pathway in processes such as developmental patterning [4], tissue interactions [5], and cell signaling through the primary cilia [6-9]. Potential benefits of obtaining a better understanding of hedgehog signaling include advancing knowledge of embryonic development [3], regeneration [10], and cancer [11].

Along with numerous other functions, hedgehog signaling is essential for the development of vertebrate epithelial appendages, such as hair, feathers, and teeth [12-14]. In humans, alteration of hedgehog signaling has been linked to diseases with dental phenotypes including solitary median maxillary central incisor syndrome [15,16] and odontogenic keratocysts [17,18]. Most of the information regarding hedgehog signaling in tooth development has come from detailed studies in the mouse [19-21]. However, expanding knowledge of the roles of hedgehog signaling during tooth development in a comparative evolutionary context has the potential to uncover more data regarding both how vertebrate teeth form during embryonic development and how they have changed during evolution.

Sites of early vertebrate tooth formation are characterized by signaling interactions between epithelial cells and nearby mesenchymal tissue [22]. The first morphological sign of tooth initiation is a thickening of the dental epithelium, followed shortly thereafter by epithelial invagination and morphogenesis, which surrounds condensing mesenchyme cells [23]. These events take place by a combination of guided cell movements, shape changes, and localized regions of proliferation [20]. Later in development, epithelial ameloblasts and mesenchymal odontoblasts secrete the organic components of enamel and dentin, respectively, to establish the form of the mature tooth [24,25]. Cell signaling is known to be required at multiple stages during these processes, but many roles have yet to be elucidated.

During a hedgehog signaling event, a hedgehog responsive cell binds secreted ligand to Patched receptors, alleviating a repressive activity of Patched on the Smoothened transmembrane protein [26]. This action in turn sets off a signal transduction cascade eventually culminating in the activation of Gli transcription factors and subsequent target gene activation [27,28]. Activity of the pathway can be modulated at several different levels including by lipid modification of hedgehog ligands [27], the secretion of extracellular inhibitors [29,30], and by repressive autoregulation mediated by the Patched receptor itself [31].

The Sonic hedgehog ligand, Shh [32], and two Patched receptors, Ptch1 and Ptch2 [33,34], are expressed in developing mouse dental tissues. Experiments inhibiting or over-activating the hedgehog pathway in mouse embryos have demonstrated several hedgehog signaling requirements during tooth development. Examples include the inhibition of hedgehog signaling after the early epithelial-thickening stage arresting mouse tooth development [35], inhibition at the bud stage resulting in malformed teeth [35,36], and later inhibition at the bell stage affecting the timing of tooth growth [37]. These studies have revealed multiple effects of the pathway on tooth development, including in cell proliferation [38,39] and differentiation [40]. However, because of the difficulty of experimentally modifying and observing very early or later tooth developmental stages in the mouse, it remains unclear whether hedgehog signaling is required for the earliest initiation of tooth development, and whether signaling continues to be necessary throughout odontogenesis.

Zebrafish produce externally-developing, optically clear embryos, and numerous experimental techniques are available for use in this species. These features make zebrafish an excellent model system in which to extend previous mouse studies and investigate hedgehog functional requirements throughout tooth development. Although the dentition of zebrafish is reduced from that of many other vertebrate species, with teeth forming only in association with the posterior ventral surface of the pharynx [41], gene expression during early odontogenesis has been found to be very similar between fish and mammals [42-45]. This similarity suggests the likelihood of common developmental mechanisms between these two types of teeth [41,42]. The zebrafish genome contains five reported hedgehog ligands and two patched receptors, including duplicated copies of Shh (*shha *= *shh *and *shhb *= *twhh*) [46-51], but potential roles of these genes during tooth development have not previously been ascertained.

In this study, we examine the expression of all known hedgehog ligands during zebrafish pharyngeal tooth development and find that similarly to the case in mammals, only *shha *is expressed in tooth germs. Consistent with this observation, embryos mutant for *shha *fail to develop any signs of tooth development. Morpholino antisense knockdown of *shha *but not *shhb *prevents mature tooth formation, also supporting the idea that *shha *alone is required for odontogenesis. Inhibition at 30 hpf with the hedgehog pathway antagonist cyclopamine allows the early tooth-related expression of *pitx2 *to occur, but blocks all other tooth-related gene expression and the morphogenesis of teeth. Additionally, application of cyclopamine during later tooth development results in disrupted tooth morphology, suggesting a requirement for hedgehog signaling even at late stages of zebrafish tooth formation. However, over-expression of *shha *using a heat shock-inducible transgenic construct has no effect on tooth formation. From these data we hypothesize that hedgehog signaling is required throughout tooth development, even at the earliest stages, but that it is not sufficient in itself to promote *de novo *tooth initiation.

## Results

### Patched receptors and *shha *are expressed in developing zebrafish pharyngeal tooth germs

**Figure 1:**
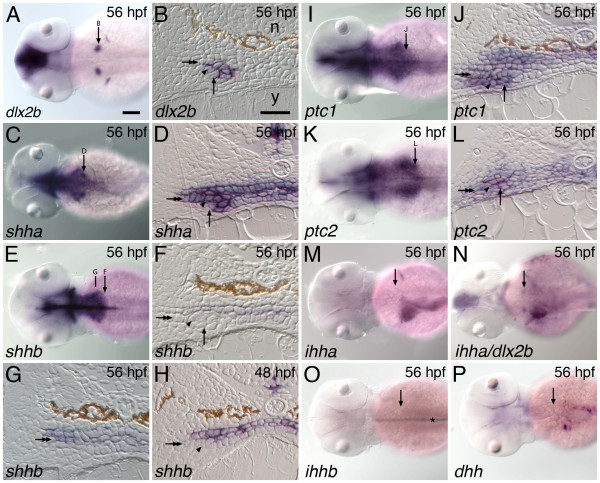
**Hedgehog pathway expression during zebrafish tooth development**. The hedgehog ligand *shha *and receptors *ptc1 *and *ptc2 *are expressed in the developing zebrafish pharyngeal dentition but we do not detect tooth-related expression of the ligands *shhb*, *dhh*, *ihha*, or *ihhb*. (A, C, E, I, K, M-P) Dorsal views of 56 hpf embryos, anterior to the left, with the location of the right-side pharyngeal tooth germ and anterior/posterior position of adjacent section indicated by an arrow. (B, D, F, H, J, L) Transverse sections of 56 hpf embryos at the level of the developing pharyngeal teeth. The lumen of the pharynx is collapsed in these histological preparations, leaving no space between the upper and lower epithelial layers. The lower pharyngeal epithelium (double arrow), dental epithelium (arrowhead), dental mesenchyme (arrow), notochord (n), and yolk (y), are indicated. (A, B) *dlx2b*, a discrete marker of zebrafish pharyngeal tooth germs, is expressed in both dental epithelium and mesenchyme [42]. Only epithelial expression is visible in B, however. (C, D) The hedgehog ligand *shha *is expressed widely in the pharyngeal epithelium including the dental epithelium, but not in the dental mesenchyme. (E, F) In contrast, at the anterior-posterior level of the developing teeth, *shhb *expression appears restricted to the upper pharyngeal epithelium and is absent from the lower epithelium and any part of the tooth germs. (G) A more rostral section from the same specimen as (F) exhibits expression in both pharyngeal epithelial layers. (H) Expression of *shhb *is also not seen in tooth germs at other stages of development such as 48 hpf when dental epithelial thickening is observed. The hedgehog receptors *ptc1 *(I, J) and *ptc2 *(K, L) are expressed widely in both the pharyngeal epithelium and in adjacent mesenchyme, encompassing both tissue layers in the tooth germs. We searched for, but were unable to detect, dental expression of the hedgehog ligands *ihha *(M; N, double label with *dlx2b*), *ihhb *(O; asterisk: notochord expression), and *dhh *(P). Scale bars: (A) 100 μm, (B) 25 μm.

We conducted a survey of hedgehog ligand and Patched receptor mRNA expression in and around developing zebrafish pharyngeal tooth germs at four-hour intervals from 36 through 56 hpf. This time period is a developmental window during which odontogenic gene expression and the early morphogenesis of the first pair of developing teeth (sometimes designated 4V_1 _[52]) have been reported to take place [42,53]. As a molecular marker of developing tooth germs for comparison, we used mRNA expression of the *dlx2b *transcription factor because it is discretely expressed in both the outer epithelium and inner mesenchyme of this earliest-developing bilateral pair of teeth starting at 48 hpf and not in the immediately surrounding tissues (Figure 1A and 1B; [42]).

Five hedgehog ligands have been identified in the zebrafish genome: *shha *(formerly *shh*), *shhb *(formerly *twhh*), *dhh*, *ihha *(formerly *hha*), and *ihhb *(formerly *ehh*, [46]). *shha *is expressed broadly in the lining of the pharynx from 36 to 56 hpf as visualized in whole-mount embryos, with two relatively darker expression domains in the position expected for pharyngeal tooth germs (Figure 1C). Sectioning 56 hpf specimens reveals that *shha *transcripts are present in both the upper and lower pharyngeal epithelial layers as well as the ventrally underlying dental epithelium (Figure 1D). Expression appears continuous between tooth-forming and adjacent epithelial regions, which is in contrast to the restriction of Shh expression to the dental epithelium seen in the oral teeth of mammals and of other fish species (see Discussion).

The pharyngeal expression of *shhb *in whole-mount specimens appears to be very similar to that of *shha *except that darker spots of expression are not seen at the location of the developing teeth (Figure 1E). In anterior regions of the pharynx, sectioning reveals that *shhb *is expressed in both the dorsal and ventral epithelia (Figure 1G). However, at the relatively posterior location where zebrafish pharyngeal teeth develop at 56 hpf, *shhb *expression appears limited to the upper pharyngeal epithelium and is absent from the ventral pharyngeal epithelium or any part of the developing tooth germs (Figure 1F). Expression is also absent from tooth germs at other developmental stages (*e.g*. 48 hpf, Figure 1H). The expression in the upper pharyngeal epithelium at this location also appears weaker than at more anterior levels from the same individuals. Thus, while whole-mount expression between *shha *and *shhb *appears similar, sections reveal that *shhb *is not expressed in developing teeth.

Two hedgehog receptors, *ptc1 *and *ptc2*, have also been identified from the zebrafish genome [49]. Both are expressed widely in the pharyngeal region, including in the epithelium and mesenchyme of developing teeth and in neighboring epithelial and mesenchymal tissues (Figure 1I-L).

We searched for, but did not find pharyngeal expression of the other known zebrafish hedgehog ligands *ihha *(Figure 1M and 1N), *ihhb *(Figure 1O), or *dhh *(Figure 1P) during any of the time points examined. However, expression domains in non-dental tissues were clearly visible including expression of *ihhb *in the notochord [54] and *ihha *in midline and left-side gut structures, likely including the liver and swim bladder primordia [55,56]. Expression of *ihha *was relatively near to the pharyngeal tooth germs, but double labels with *dlx2b *revealed no overlap (Figure 1N).

### *shha *is necessary for tooth development

**Figure 2:**
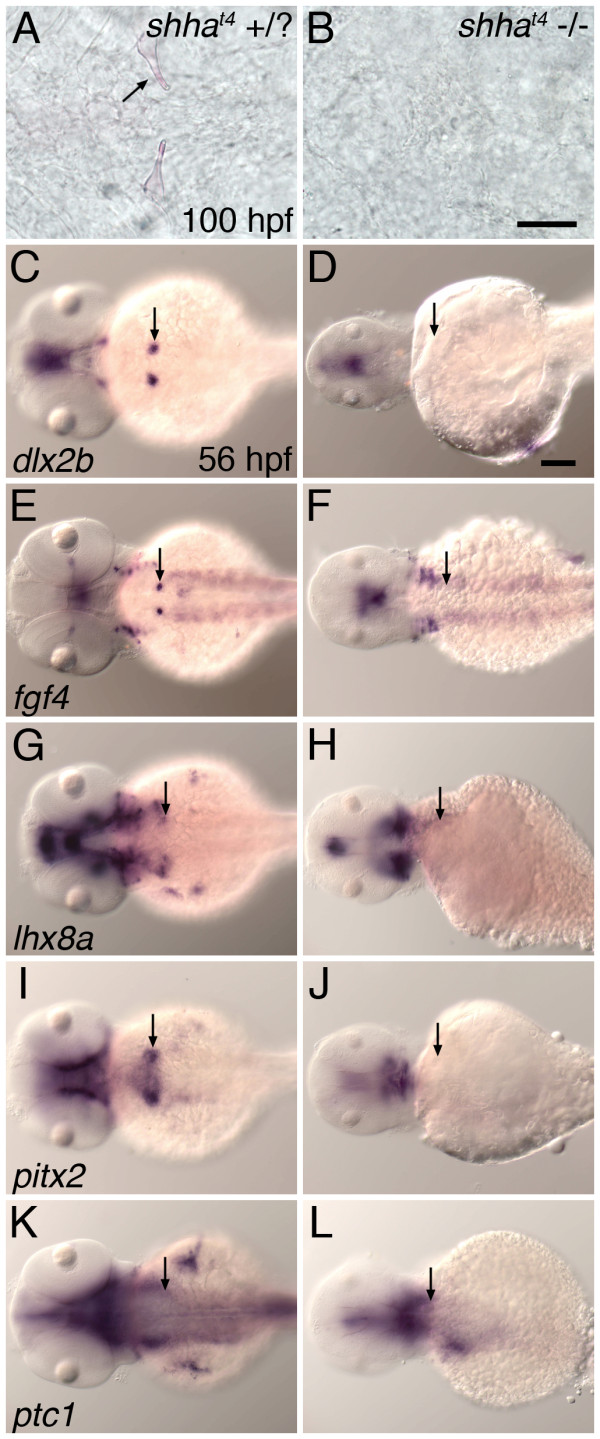
**Zebrafish mutant for *shha *do not exhibit tooth-related developmental gene expression and do not form mature teeth**. (A, B) Ventral views of the pharyngeal region at 100 hpf stained with alizarin red to highlight calcified structures. Teeth appeared normal in wild-type and *shha^t4 ^*heterozygous individuals (A), but were not detected in *shha^t4 ^*homozygous mutant siblings (B). mRNA *in situ *hybridization analysis at 56 hpf demonstrated that pharyngeal tooth-related expression of *dlx2b *(C, D), *fgf4 *(E, F), *lhx8a *(G, H), and *pitx2 *(I, J) was completely absent from homozygous mutant embryos but present in sibling controls possessing at least one wild type *shha *allele. Expression of *ptc1 *in the tooth-forming region appeared to be maintained in all genotypes (K, L). (C-L) Dorsal views, anterior to the left, position of right side tooth germs indicated (arrows). Scale bars: (B) 50 μm, (D) 100 μm.

Because of the dental expression of *shha*, we were interested in testing the necessity of this gene in tooth development by examining embryos from the *shha^t4 ^*mutant line, which is completely deficient in Shha protein [57] (Figure 2). We found that homozygous mutant embryos lacked any sign of mature teeth at 100 hpf (n = 8; Figure 2B). Looking earlier in development, while mRNA expression of the receptor *ptc1 *was present in the pharyngeal region of mutant embryos, the expression of several tooth germ marker genes was completely missing from both the dental epithelium and mesenchyme. These genes included *dlx2b *(n = 4); *fgf4*, a marker of a subset of the dental epithelium [42] (n = 5); the dental mesenchyme marker *lhx8a *(formerly *lhx7*, n = 6); and *pitx2*, a gene expressed broadly in pharyngeal epithelium and tooth germs (n = 5) (Figure 2C-L). The absence of *pitx2*, the earliest reported marker of tooth initiation in both fish and mammals [42,58], suggests that *shha *is required at a very early stage in the initiation of tooth development, although we can not rule out even earlier indirect effects on pharyngeal morphogenesis.

**Figure 3:**
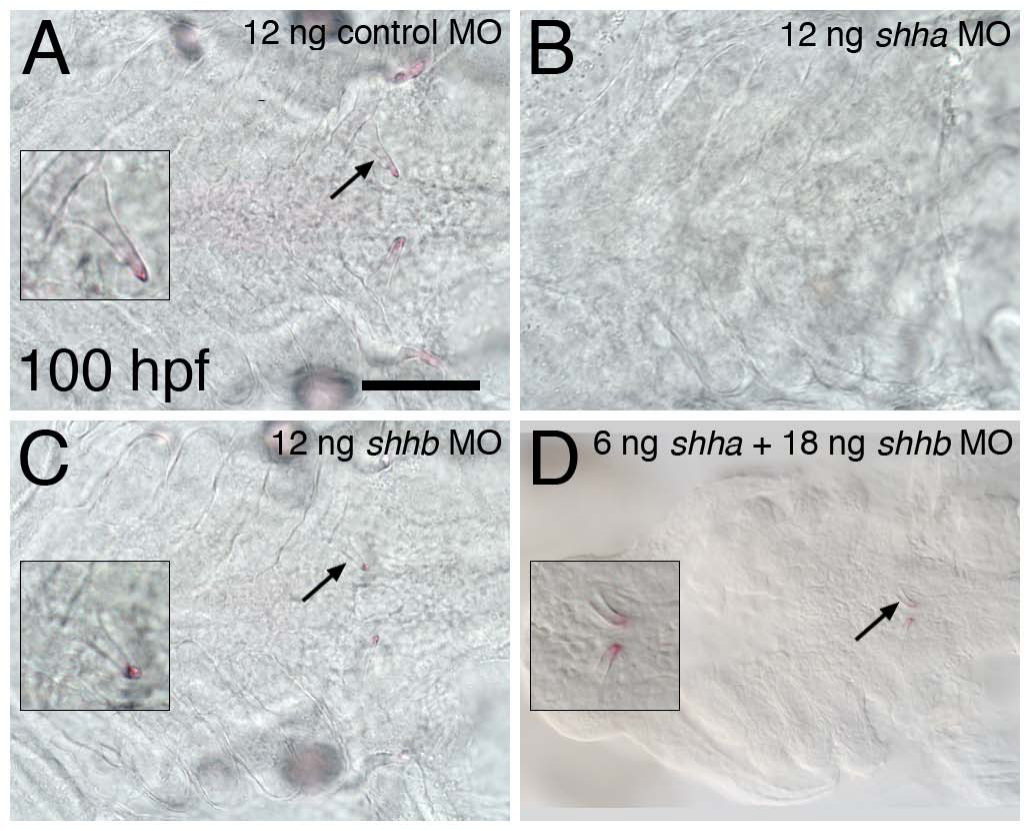
**Antisense RNA inhibition of *shha*, but not *shhb*, prevents the formation of mature teeth**. Ventral views of the tooth-forming region of alizarin red stained 100 hpf embryos. Teeth develop normally in control morpholino injected embryos (A, left-side tooth shown in inset). Teeth are eliminated from *shha *MO injected embryos (B), but retained in embryos injected with an identical amount (12 ng) of the *shhb *morpholino (C). The combination of 6 ng *shha *MO with 18 ng *shhb *MO results in teeth forming closer to the midline and appearing slightly developmentally delayed but otherwise with normal morphology (D). Scale bar 100 μm.

Based upon our expression analysis, we hypothesized that *shha *but not *shhb *would be necessary for tooth development. To test this idea directly, we injected antisense morpholino oligonucleotides (MOs) into embryos to knock down the function of *shhb*. At MO concentrations that eliminated teeth in *shha *MO injected embryos (12 ng, n = 6), *shhb *MO injected embryos developed teeth morphologically similar to those of controls (n = 7, Figure 3). To examine possible combinatorial effects between these two genes, we injected a suboptimal dose of *shha *MO (6 ng) along with a larger amount of *shhb *MO (18 ng). The resulting embryos exhibited the additive phenotypes of this double knockdown that have previously been described including reduced head size and midline defects [59]. However, except for teeth forming closer to the midline and appearing delayed relative to control embryos, they otherwise appeared relatively normal (n = 8, Figure 3D). These results are consistent with the idea that *shhb *is indeed not required for zebrafish pharyngeal tooth development but does not rule out the possibility that *shhb *may normally modulate tooth development in some way, especially at later stages.

### Hedgehog signaling is required at multiple stages of tooth development

**Figure 4:**
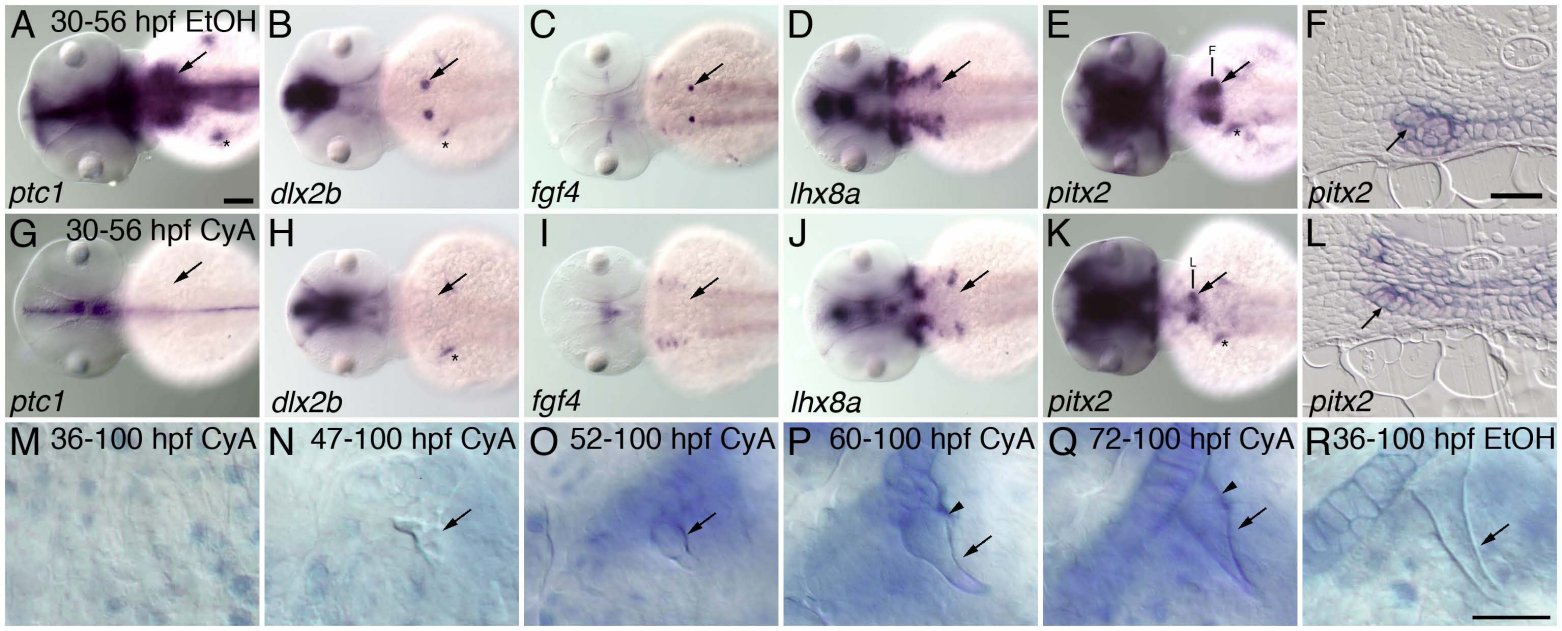
**Hedgehog requirements throughout tooth development**. Inhibition of hedgehog signaling with cyclopamine (CyA) starting at 30 hpf prevents tooth morphogenesis, and treatments at subsequent stages reveal later hedgehog signaling requirements during tooth formation. (A-E) mRNA *in situ *hybridizations of pharyngeal tooth gene expression at 56 hpf for *ptc1 *(A), *dlx2b *(B), *fgf4 *(C), *lhx8a *(D), and *pitx2 *(E) in control embryos exposed to 0.5% EtOH from 30-56 hpf (dorsal views, anterior to the left, right side tooth germs indicated with an arrow, asterisks designate pectoral fin or girdle expression). (G-K) Embryos exposed to 50 μM CyA and 0.5% EtOH from 30-56 hpf show severely reduced or absent pharyngeal tooth expression of *ptc1 *(G), *dlx2b *(H), *fgf4 *(I), and *lhx8a *(L); but *pitx2 *(K) expression is maintained. (F, L) Transverse sections through the pharyngeal tooth forming region of 56 hpf embryos treated from 30 to 56 hpf in 0.5% EtOH with and without 50 μM CyA. Dental epithelial morphogenesis is highlighted by *pitx2 *mRNA expression in control embryos (F, arrow), but after CyA exposure the lower margin of the pharyngeal epithelium lacks the thickening or curved appearance characteristic of early tooth morphogenesis (L, arrow). (M-R) Right-side first pharyngeal tooth (arrows) in CyA or control treated, alcian blue stained and cleared 100 hpf larvae. No mature tooth formation was visible when CyA treatment was begun by 36 hpf (M). CyA exposure from 47 hpf allowed some limited mineralized morphogenesis, but it severely disrupted the shape of the entire tooth, causing it to appear unorganized (N). Treatment from 52 hpf resulted in teeth with a more regular appearance, but somewhat small and rounded (O). 60 hpf (P), and 72 hpf (Q), treatment resulted in teeth with only the later-developing shaft and base of the tooth having an abnormal rounded morphology (arrowhead) relative to controls (R). Scale bars: (A) 100 μm, (F) and (R) 25 μm.

To test possible later functions of hedgehog signaling in zebrafish pharyngeal tooth development we exposed embryos to cyclopamine (CyA), a compound that inhibits hedgehog signaling by binding to the Smoothened transmembrane protein [60,61]. We chose 30 hpf as the time point of initial CyA exposure because treatment at this stage allows early development to proceed normally, but is still 6 hours earlier than *pitx2 *expression initiates in developing zebrafish tooth germs [42], 14 hours before expression of other markers of tooth development is present [42,62], and 18 hours before the first morphological sign of tooth development [53]. After continuous exposure to 50 μM CyA starting at 30 hpf, we found that the tooth-related expression of several genes was severely reduced or eliminated by 56 hpf, including *ptc1 *(Figure 4A, G n = 11), *dlx2b *(Figure 4B, H n = 10), *fgf4 *(Figure 4C, I n = 11), and *lhx8a *(Figure 4 D, J n = 11). Patched has been shown in certain cases to require Shh signaling for the maintenance of its expression [50], thus the downregulation of *ptc1 *after cyclopamine treatment was expected independent of any effects on tooth development. However, the elimination of other tooth-related gene expression is suggestive of early tooth developmental arrest. In contrast to the results with the above-mentioned markers, expression of *pitx2 *appeared reduced but was always present after CyA treatment starting at 30 hpf (Figure 4E, K n = 11), suggesting that some hedgehog signaling involved with tooth initiation takes place before this developmental time point. However, sections reveal that while *pitx2 *expression is initiated, there is no evidence that dental morphogenesis takes place (Figure 4F, L n = 4).

We also examined hedgehog signaling requirements during later stages of tooth morphogenesis by applying CyA at progressively more advanced developmental stages and scoring dental phenotypes in whole-mount histological preparations (Figure 4M-R). Exposure of zebrafish embryos to 50 μM CyA as late as 36 hpf completely eliminated mature tooth formation (Figure 4 M, n = 15). Somewhat later treatments starting at 47 hpf allowed for some tooth mineralization, but when it occurred, teeth were small and misshapen (Figure 4N, n = 10). CyA application at subsequent time points (52 hpf, Figure 4O, n = 15; 60 hpf, Figure 4P, n = 21; and 72 hpf, Figure 4Q, n = 19) allowed progressively more complete tooth development, but tooth morphology was abnormal relative to controls (Figure 4R). We noted that the shafts and bases of mineralized teeth appeared to be more severely effected than the tips, consistent with cyclopamine interfering with later stages of the normal tip-to-base progress of tooth formation. Together, these CyA inhibition data suggest that hedgehog signaling is required both at the initiation of dental development and during later tooth morphogenesis.

### Over-activation of hedgehog signaling has no observable effect on tooth development

**Figure 5:**
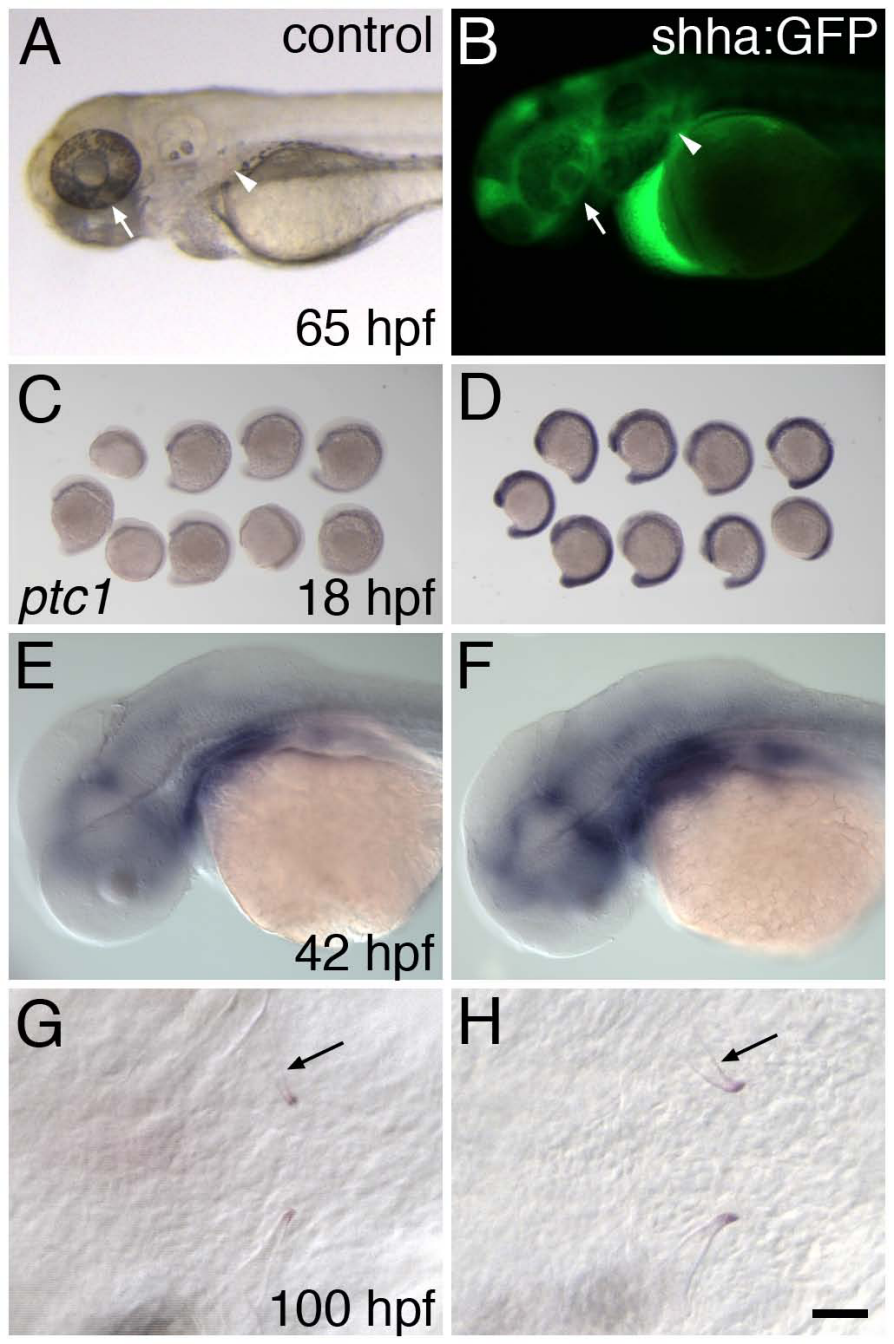
**Overexpression of *shha *does not alter tooth formation or morphology**. (A-F) Control *shha *overexpression experiments. After heat-shock at 12 hpf, 65 hpf control embryos display normally-developing retinas (A, arrow) whereas the ventral retinas of shha:GFP injected siblings is severely reduced (B, arrow). While absent from controls, GFP expression is visible in shha:GFP injected embryos, including in the region where teeth are forming (B, arrowhead), and appears to be concentrated in extracellular spaces. (C) *ptc1 *mRNA expression in uninjected embryos at 18 hpf subsequent to a heat shock at 12 hpf. (D) Embryos injected with the shha:GFP construct undergoing the same heat shock treatment exhibit strong upregulation of *ptc1*. Similarly, relative to controls (E), shha:GFP injected embryos showed increased *ptc1 *expression at 42 hpf after heat shock at 36 hpf (F). However, *shha *overexpression at neither stage altered mineralized tooth shape (arrow) or timing of tooth development as visualized at 100 hpf (G, H). Scale bar 25 μm.

We also investigated whether the upregulation of hedgehog signaling has an effect on zebrafish tooth initiation or morphogenesis. To this end we produced and injected a DNA construct consisting of a Hsp70 heat-shock inducible promoter driving the expression of a zebrafish shha:GFP fusion protein. Injection of the construct at the 1-cell stage and subsequent heat-shock at 12 hpf produced strong GFP expression (including in the tooth-forming region; arrowheads, Figure 5A-B), previously described ventral eye malformations [63], and upregulation of *ptc1 *mRNA expression by 18 hpf (n = 9, Figure 5C-D). Upregulation of zebrafish *ptc1 *by *shha *has been previously reported [50] and suggests that the shha:GFP construct has activity *in vivo*. Similar experiments with *shha *over-expression induced at 36 hpf also resulted in *ptc1 *upregulation by 42 hpf (Figure 5E-F). However, tooth morphogenesis appeared normal and teeth formed at approximately the normal developmental rate after injected embryos were heat-shocked at 12 hpf (Figure 5G-H), and the same was true whether heat shocks were applied at 24 hpf, 36 hpf, or every four hours from 18-100 hpf (not shown). These experiments suggest that increasing the expression of *shha *beyond the normal level has little or no effect on tooth development.

## Discussion

### Hedgehog requirements for early tooth developmental initiation

Previous research in other species has been inconclusive regarding whether hedgehog signaling is necessary for the earliest events in the initiation of tooth development. In mice, this line of investigation has been limited both due to essential roles of hedgehog signaling in early development and problems with the accessibility of developing mammalian embryos. In a Shh knockout mouse mutant line, homozygous mutant embryos fail to develop mandibular and maxillary arches, precluding potential tooth phenotypes from being examined [64]. A mouse strain was also created containing a conditional deletion allele of Shh [36]. However, in this conditional knockout line Shh is not inactivated until after embryonic day 11 (E11), more than a day after Shh expression commences in mouse oral epithelium, and at a time when tooth morphogenesis is already visible [65]. A similar conditional knockout allele of Smoothened was also investigated but subject to the same limitation in the timing of inactivation [39]. Thus due both to the nature of the conditional inactivation alleles available and to early requirements in oral-facial development, studies involving mouse mutants have been unable to assess hedgehog function in the initiation of tooth development.

A variety of other techniques for inhibiting hedgehog function during mouse tooth development have been employed but have been similarly limited in their success at affecting very early events. Application of jervine, an alkaloid inhibitor of hedgehog signaling [66], to mice developing *in utero *was seen to block incisor formation [65]. However, similarly to the mouse Shh mutant line, this jervine result may have been due to the general inhibition of distal jaw development where these teeth normally form. In an alternative to *in utero *experimentation, mouse mandibular explant cultures in which developing tooth germs can be experimentally modified have been made, with stages starting as early as E10.5. Inhibiting hedgehog signaling with a Shh blocking antibody or with the indirect hedgehog antagonist forskolin in mandibular explants at this stage causes tooth developmental arrest at the epithelial thickening stage [35]. However, in each of these experiments some sign of tooth developmental initiation takes place. This observation suggests that either hedgehog signaling is not required for tooth initiation or that signaling has not been disrupted early enough in these experiments to reveal the requirement.

In other (non-zebrafish) species, hedgehog pathway mutants are not available for analysis. However, hedgehog ligand expression during embryonic tooth development has begun to be examined in diverse species including a shark [67], teleost fishes [68-70], a lungfish [71], and reptiles [72,73]. Importantly, in several of these studies hedgehog loss of function analysis has been performed using the small-molecule chemical inhibitor cyclopamine. In cichlid fishes, cyclopamine treatment has been shown to reduce the numbers of developing tooth germs but does not completely block all odontogenesis [68]. In squamate reptiles, cyclopamine treatment has been shown to arrest tooth development at the early epithelial thickening stage in snakes [73], and blocks tooth morphogenesis in lizards [72]. These results suggest that as with the experiments in mice, hedgehog inhibition with cyclopamine does not completely inhibit tooth development in other vertebrate species. However, questions remain regarding whether the timing of cyclopamine application in these experiments or other factors may have prevented the complete inhibition of hedgehog signaling at the necessary stages to disrupt tooth formation. In contrast, our analysis of the zebrafish *shha *mutant suggests that hedgehog signaling is essential for the earliest stages of tooth initiation. This idea is supported by the complete absence of tooth-related gene expression in *shha *homozygous mutant embryos, including the marker *pitx2*, which is the earliest marker of tooth formation in both fish and mammals [42,58] (Figure 2).

One possible reason why the zebrafish *shha *mutant phenotype may be more interpretable than the dental phenotypes of *Shh *knockout mice is because of the different location in which zebrafish form teeth. Mouse teeth are dependent upon the craniofacial prominences that form the jaws developing to a certain extent in order to have a place in which to form (*e.g*. [74]). Consequently, dental phenotypes may be obscured when craniofacial morphogenesis is disrupted at an early stage. In zebrafish *shha *mutants, the pharyngeal region appears to develop relatively normally, presumably giving pharyngeal teeth a place in which to form should they be capable of doing so. Another possible explanation for why the zebrafish posterior pharyngeal region may develop relatively normally in *shha *mutant embryos is that the expression of other hedgehog ligands, notably *shhb *and *ihha*, may be able to compensate for the lack of *shha *expression in the developing pharynx (Figure 1). This idea is supported by the observation that *ptc1*, which is normally upregulated in cells receiving a hedgehog signal [49], continues to be expressed in the pharyngeal region of *shha *mutant embryos (Figure 2L), but that *ptc1 *expression appears severely reduced after treatment with cyclopamine (Figure 4G). It follows that global inactivation of hedgehog signaling induced by cyclopamine may limit the usefulness of this compound in assessing hedgehog requirements at tooth initiation in many species. If hedgehog signaling is completely inactivated early enough, the oral and pharyngeal regions in which teeth form may not develop to the extent where they are capable of harboring tooth germ development, resulting in a situation similar to mouse *Shh *knockout mutants where tooth phenotypes are not assessable.

### Hedgehog requirements during later tooth morphogenesis

Hedgehog signaling requirements during later tooth morphogenesis have been tested at certain developmental stages in a few species, but these experiments have been limited by the embryonic stages in which tooth germs are accessible and are able to be manipulated experimentally. For instance in the mouse, hedgehog inhibition during late tooth stages is restricted by the incomplete penetration of compounds such as jervine *in utero *[65], the timing when conditional alleles can be activated [75], or by the stages that are possible to access when performing mandibular explants [76]. With mandibular explant experiments, it is necessary to re-implant experimentally-manipulated tooth germs into a host environment for complete growth [19], which limits both the stages at which signaling can be blocked and the subsequent observation of developmental phenotypes.

Examples of late stage hedgehog inhibition include a study in mice in which relatively late E17 bell stage tooth germs in tissue culture were injected with a Shh-blocking antibody, and growth was assessed after re-implantation into host tissue [37]. Developmental delay was observed after this treatment, but no sign of altered dental morphogenesis or cell differentiation was noted. Similar experiments were performed in lizard and snake species, where tissue explants were exposed to cyclopamine and morphological changes to developing tooth germs were observed [72,73]. Alteration in developing tooth germ morphology was observed after hedgehog inhibition in these experiments. However, later assessment of tooth shape could not be observed, possibly due to the relatively short length of time tissues will continue to grow normally in culture.

Our experimental inhibition of hedgehog signaling with *in vivo *cyclopamine treatment at 30 hpf appears to arrest zebrafish pharyngeal tooth development just after initiation (Figure 4). In CyA treated embryos, we continue to see localized expression of the dental epithelial marker *pitx2*, but no expression of other markers of developing tooth germs, and no sign of tooth morphogenesis. We interpret the maintenance of *pitx2 *expression in the region where pharyngeal teeth would normally form in these CyA-treated embryos to indicate that at least some early part of tooth initiation has taken place by 30 hpf, but that tooth development has been arrested soon after this stage. This result could be consistent with those seen in mice [36,65,66,75,76], other fish species [67,68], and in reptiles [72,73] where hedgehog inhibition allows tooth initiation but arrests development soon afterwards. Coupled with the complete loss of tooth development we report in *shha *mutant embryos, this could also be further indication that the inability of arresting tooth development by blocking hedgehog signaling in other species may have more to do with the developmental stage of inhibition or indirect effects of reducing hedgehog function on supporting tissues rather than differing requirements for early hedgehog function in tooth development.

An advantage to working with a species like zebrafish where chemical inhibitors penetrate unmodified embryos is that this makes straightforward the testing of hedgehog signaling roles at a variety of developmental stages. With later-stage zebrafish cyclopamine treatment, we saw disrupted tooth morphology at all stages of tooth development investigated (Figure 4). We suggest that these results indicate a continuous requirement for hedgehog signaling throughout tooth morphogenesis, possibly including for roles in cell differentiation, proliferation, and matrix secretion. These results are in contrast to the report of relatively normal morphology after hedgehog inhibition at the bell stage in mice [37]. However, it remains to be seen whether this apparent difference in activity between late dental hedgehog inhibition in mouse and zebrafish is the result of alternative experimental methods, a difference between mechanisms of oral and pharyngeal tooth development, or an evolutionary difference between hedgehog signaling requirements in late tooth development between fish and mammals.

Given the importance of hedgehog signaling for even the earliest events in tooth development, a corresponding question becomes whether a hedgehog signal is sufficient to induce tooth development. In other vertebrates, this question has been investigated using Shh protein coated bead implantation. Shh over-expression by this method has been shown to increase cell proliferation in developing tooth germs in both mice and snakes [72,76]. In another study, implanting Shh coated beads in mouse embryos E10.5 stimulated tooth morphogenesis, causing nearby ectopic epithelial invaginations and mesenchyme condensation with associated tooth specific gene expression [77]. In contrast, a similar study found no effect of Shh bead implantation on either tooth cytodifferentiation or cusp number [40]. In a fourth example in mice using a different technique, Shh over-expression driven by the K14 epithelial promoter inhibited cell proliferation but arrested tooth development at bud stage [78]. It is unclear whether these seemingly contradictory results are the result of differing techniques, stages of treatment, or phenotypic assessment.

In our similar transgenic experiments in zebrafish, when we over-expressed *shha *using a heat shock promoter, we saw neither a stimulation nor repression of tooth development, regardless of the timing of overexpression. We demonstrated *in vivo shha *activity by examining *ptc1 *activation, but this over-expression of *ptc1 *may represent more than just a control. Over-expression of *ptc1 *is predicted to have an inhibitory effect on the activity of the hedgehog pathway [31], and thus this extra expression may buffer the effects of additional Shh [51,79]. High enough levels of Shh might be expected to overpower this buffering ability of *ptc1*, which could potentially explain why dental phenotypes were seen in mouse experiments using Shh coated beads.

The expression of *shha *in the pharyngeal epithelium immediately adjacent to the pharyngeal tooth germs could also potentially be involved with suppressing a zebrafish Shh over-expression dental phenotype. A notable difference between mouse and zebrafish tooth-related Shh expression is that in mouse, Shh expression becomes restricted to developing tooth germs and eliminated from surrounding tissues [65,80], whereas in the zebrafish, expression adjacent to the tooth germs is maintained. In mouse, localization of Shh protein has been proposed to play a role in positioning where tooth germs will form [80]. The maintenance of adjacent Shh expression in zebrafish tissues next to developing tooth germs suggests that either the localization of Shh to the tooth germ may not be necessary for proper tooth germ positioning, or that Shh may have a different function in this regard between fish and mammals. Zebrafish lack oral teeth [70], but where fish species that possess oral teeth have been examined, Shh expression is first widespread in the oral epithelium before becoming restricted to tooth germs as in mouse [43,70]. One possible explanation for the broad pharyngeal *shha *expression in zebrafish is that additional mechanisms may be suppressing induction of tooth development in adjacent regions: mechanisms that would not be required in the oral region. Secreted hedgehog pathway inhibitors have been characterized in other systems [30] and future comparative studies of epithelial gene expression could reveal candidates for putative pharyngeal tooth repressors.

### Evolution of the dental hedgehog pathway

We examined the mRNA expression of all known zebrafish hedgehog pathway ligands in the developing pharyngeal dentition, partly due to an interest in learning whether hedgehog ligand use has changed during the evolution of vertebrate tooth development. In the mouse, only the Shh ligand is expressed during tooth development [32,80]. However, zebrafish, as teleosts, are hypothesized to have undergone a genome duplication event not shared with mammals [81,82], and possess duplicate copies of both the Shh and Ihh ligands [46-48,50]. The Duplication, Degeneration, and Complementation (DDC) model [83], proposes that the primary way that duplicated developmental genes are preserved in a lineage is by the complementation of their functions, often by loss of different developmental expression pattern domains between duplicate pairs. The very dissimilar and non-overlapping expression patterns of zebrafish Ihh duplicates could potentially fit this model (Figure 1G, H). Additionally the non-overlapping expression of *shha *and *shhb *in the posterior pharynx (Figure 1) as well as the different functions in tooth development as evident by our *shhb *inhibition result also is consistent with this model. The organization of genomic regions regulating Shh expression has begun to be characterized in mice [84], and it will be interesting as the zebrafish genome is similarly mapped, to identify the *cis*-regulatory regions responsible for the differences in expression of *shha *and *shhb*. One prediction would be that enhancers responsible for driving *shhb *in the posterior pharynx and teeth have accumulated mutations or been lost altogether, removing this duplicate from functioning in fish tooth development and fixing *shha *in this role.

Lastly, the difference in Shh expression between developing oral teeth in species that possess them and zebrafish pharyngeal teeth regarding whether Shh is expressed in adjacent epithelial tissues has interesting evolutionary implications. Might the oral/pharyngeal Shh expression difference represented ancient and distinctly different mechanisms of hedgehog action in tooth development? The evolutionary relationship of oral and pharyngeal teeth from the fossil record has been controversial regarding whether they evolved simultaneously, independently, or one from the other [85-87]. One interpretation of the similarities and differences in oral/pharyngeal Shh expression is that all teeth share a deep single evolutionary origin but have undergone considerable independent evolution since, which may be consistent with a scenario where one kind of tooth evolved from the other [88]. Future comparative studies of Shh protein localization enhanced with experiments to test the function in both the oral and pharyngeal regions of representative vertebrate species will help resolve this issue.

## Conclusions

In this study we have presented data that suggest that the hedgehog signaling pathway ligand *shha *and receptors *ptc1 *and *ptc2 *are expressed during zebrafish tooth development but that the ligands *shhb*, *dhh*, *ihha*, and *ihhb *are not. Consistent with these expression data, functional analysis using morpholino mRNA inhibition suggests that only the *shha *ligand is necessary for tooth development. By examining a zebrafish line mutant for *shha *and by using the hedgehog pathway inhibitor cyclopamine, we conclude that hedgehog signaling is required for proper tooth development from the earliest stages of tooth initiation all the way through mineralized tooth morphogenesis. However, over-expression of *shha *has no observable effect on tooth development. Together with previous work in other species, these results suggest that the function of hedgehog signaling may be largely conserved in vertebrate tooth development, but several interesting evolutionary questions remain to be explored further.

## Methods

### Fish strains

Wild-type strains of zebrafish (*Danio rerio*) were maintained as previously reported [42]. Developmental stages are stated in hours post fertilization (hpf) as in [89]. The zebrafish *shha^t4 ^*mutant line [57] was obtained from the Zebrafish International Resource Center. All animal experiments were conducted according to protocols approved by the Institutional Animal Care and Use Committees at Bowdoin College (2008-16, 2008-17) and the University of Colorado (06-07-STO-01, 06-07-STO-02, 08-07-STO-01, 08-07-STO-02).

### Gene expression

Messenger RNA *in situ *hybridization, whole-mount viewing, and sectioning were performed as previously described [42]. Gene nomenclature is coordinated with that used in the Zfin database [46]. The source sequences for antisense probes were as follows: *dlx2b *[90]; *lhx8a *(formerly *lhx7*), *pitx2 *[42]; *shha *(formerly *shh*) [70], *shhb *(formerly *twhh*) [48]; *ptc1*, *ptc2 *[49]; *ihhb *(formerly *ehh*) [54]; *ihha *(sequences 256-1537 of Genbank accession number NM_001034993); and *dhh *(48-883 of DQ066429).

### Functional analysis

For cyclopamine (CyA) experiments, embryos were exposed to a concentration of 50 μM CyA in 0.5% EtOH and 30% Danieu's medium [91]. Control embryos were exposed to 0.5% EtOH and 30% Danieu's medium for equal time periods. Antisense knockdown of *shha *and *shhb *transcripts were done with the morpholino oligonucleotide (MO) sequences described in [59]. The standard control MO (Gene Tools, Philomath, OR) targets a human β-hemoglobin gene. To introduce the MO into embryos, approximately 6, 12, or 18 ng of MO in a 0.2 M KCl and 0.2% phenol red solution was injected into the yolk of 1-cell stage embryos.

Overexpression of *shha *was obtained using a hsp70:shha:GFP plasmid, a description of which was reported in [92]. In preparation for injection, 250 ng of plasmid DNA was digested with 5 U I-SceI homing endonuclease (New England BioLabs) for 1 hour at 37°C, mixed 1:1 with 0.2 M KCl and 0.2% phenol red, and stored at -20°C. Approximately 1 nl (12 pg DNA) of this solution was injected into the yolk of 1-cell embryos. Heat shock overexpression was induced by placing dishes of embryos in an EcoTherm programmable incubator (Torrey Pines Scientific, San Marcos, CA) and setting it to change the temperature to 40°C for 30 minutes at various time points.

### Microscopy

Embryos were photographed using bright field or differential interference contrast optics on a Zeiss Axiovert 135 compound microscope using an AxioCam digital camera (Zeiss, Thornwood, NY), a Leica MZ16F stereomicroscope with DFC300FX camera, or a Leica DMI3000B inverted scope with DFC420C camera (Leica Microsystems, Bannockburn, IL). Photograph files were processed with Adobe Photoshop (Adobe Systems, San Jose, CA) and the GNU Image Manipulation Program http://www.gimp.org.

## Authors' contributions

WJ and DS conceived of and designed the study, carried out the expression and inhibition experiments, and drafted the manuscript. JY carried out the over-expression experiments and helped draft the manuscript. All authors read and approved the final manuscript.
